# The COVID-19 Pandemic, Social Ties, and Psychosocial Well-Being of Middle-Aged Women in Rural Africa

**DOI:** 10.1177/23780231231171868

**Published:** 2023-05-20

**Authors:** Victor Agadjanian

**Affiliations:** 1 University of California-Los Angeles, Los Angeles, CA, USA

**Keywords:** COVID-19 pandemic, social ties, psychosocial well-being, women, sub-Saharan Africa

## Abstract

The study contributes to the understanding of the societal impact of the coronavirus disease 2019 pandemic in the Global South by examining longer term implications of pandemic-induced disruptions and deprivations for social ties and psychosocial well-being. Using data from a survey of middle-aged women in rural Mozambique, the author finds a negative association between the pandemic-triggered household economic decline and perceived changes in the quality of relations with marital partners, non-coresident children, and relatives, but not with generally more distant actors, such as coreligionists and neighbors. In turn, multivariable analyses detect a positive association of changes in the quality of family and kin ties with participants’ life satisfaction, regardless of other factors. Yet women’s expectations for changes in their household living conditions in the near future show a significant association only with changes in the quality of relations with marital partners. The author situates these findings within the context of women’s enduring vulnerabilities in low-income patriarchal settings.

The coronavirus disease 2019 (COVID-19) pandemic and its epidemiological consequences and social fallouts globally have generated a massive and growing volume of scholarship. In sub-Saharan Africa, although the scale of infection and mortality caused by severe acute respiratory syndrome coronavirus-2 infection have been debated because of limited and questionable statistics (Maeda and Nkengasong 2021; Okonji et al. 2021), the indirect effects of the pandemic and of the societal disruptions that it triggered on various aspects of health and well-being have been widely documented. As in other part of the Global South, these effects include job and income losses and resulting poverty and nutritional insecurity (Bargain and Aminjonov 2021), as well as reduced access to schooling and to health services (Bwire et al. 2022; Hedstrom et al. 2021; Nachega et al. 2021; Perofsky et al. 2022). Yet it has been also noted that the evidence on these effects is not uniform and varies across generational, gender, and other divides (e.g., Davey et al. 2020; Espi-Sanchis, Leibbrandt, and Ranchhod 2022; Soko et al. 2021), paralleling the evidence from high-income contexts, in which the negative impact of the pandemic-related disruptions has varied by gender (e.g., Connor et al. 2020; Yavorsky, Qian, and Sargent 2021) and across ethnic and racial groups (e.g., Gauthier et al. 2020; Yaya et al. 2020), further amplifying existing disparities and inequalities.

Scholars have paid considerable attention to negative implications of pandemic-induced containment policies for mental health (for a review see, e.g., Penninx et al. 2022), even though the corresponding research in sub-Saharan Africa remains relatively scarce and largely limited to more developed countries such as the Republic of South Africa (De Man et al. 2022; Posel, Oyenubi, and Kollamparambil 2021). In high-income contexts, studies have argued that lockdown measures and related restrictions increased depression, anxiety, and loneliness (Dahlberg 2021; Killgore et al. 2020; O’Donnell et al. 2022; Prati and Mancini 2021), and these trends were especially pronounced among older adults (Dahlberg 2021; Krendl and Perry 2020; van Tilburg et al. 2020) and among women (Fiorenzato et al. 2021).

Some research in high-income contexts has also looked at the at pandemic-related “relational vulnerability” (Furfaro et al. 2021), as much of the mental health impact of the pandemic is linked to social detachment and isolation (Douglas et al. 2020). Yet it has been also argued that the pandemic has had conflicting and countervailing effects on social relations, causing both disruption and cohesion (Gauthier et al. 2020; Gupta et al. 2021). And it has been also shown that social connectedness may mitigate the negative effects of the pandemic and related containment measures on mental health (Kovacs et al. 2021; Nitschke et al. 2021; O’Donnell et al. 2022). Family-based ties and similarly close relationships have been most consequential for individual well-being in buffering the negative effects of the pandemic, especially among disadvantaged population segments (Choi, Tessler, and Kao 2022; Jace and Makridis 2021; Prime, Wade, and Browne 2020; Shepherd 2022). This evidence largely aligns with the broader research asserting the association of social networks with health behavior and outcomes (Perkins et al. 2015) and, in particular, the benefits of social connectedness for mental health (Chuang, Chuang, and Yang 2013; Ehsan and De Silva 2015). Yet as an analysis of Cacioppo, Fowler, and Christakis (2009) reminds us, social connections, however large, diverse, and intense, may not necessarily protect from loneliness and similar psychosocial challenges.

As in many other research fields and areas, sub-Saharan Africa, especially its rural parts, has been grossly underrepresented in this cross-national research, and I contribute to filling this gap by focusing on pandemic-related changes in the quality of social ties and their possible implications for psychosocial well-being in a typical rural sub-Saharan setting. My contributions to the extant scholarship, however, go beyond simply expanding its geography. First, guided by the evidence on age- and gender-specific consequences of the pandemic, I focus on the experience of middle-aged women. In highly patriarchal, impoverished sub-Saharan settings, where rural women’s control over productive and material assets is greatly constrained and their access to formal social safety nets is very limited or even nonexistent, social ties with kin and nonkin are crucial determinants and mechanisms of women’s well-being as they transition through midlife and into old age. Second, unlike studies that use clinical mental health indicators, I look at more holistic measures of subjective well-being: life satisfaction and optimism. These two outcomes are understandably interrelated, yet they capture sufficiently different dimensions of the complex panoply of individuals’ cognitive and affective evaluations of their current and prospective realities. Although these measures do not capture the infinite nuances of navigating routine and extraordinary challenges, they are indicative of broader quality of life and have shown strong connections to various aspects of physical and mental health and, accordingly, have been increasingly advocated as key measures and instruments of intervention programs and policies, especially those targeting the aging population segment (e.g., Helliwell, Layard, and Sachs 2016; Kim et al. 2021). Third, I look at what can be considered longer term implications of the pandemic. Much of the global research on psychosocial consequences of the COVID-19 pandemic has typically addressed more immediate or short-term effects of the pandemic-related containment and restrictions measures and related disruptions (e.g., Kumar and Nayar 2021; Padilla-Frausto, Pereira, and Valdivia 2022; Pfefferbaum and North 2020). Longer term effects of such measures (i.e., after they have been officially terminated but still prominently figure in societal memory and individual consciousness), have not been systematically examined. Importantly, although specific and immediate mental health reactions to pandemic-induced societal shocks, such as anxiety, depression, or loneliness, may diminish as the direct effects of such shocks abate, the longer term impact of those shocks on general psychosocial well-being may endure. Moreover, these longer term consequences may vary across different population groups, adding to existing group-specific vulnerabilities and disadvantages.

## Conceptualization

I start by examining perceived changes in the quality of rural middle-aged women’s social relations in the context of socioeconomic pressures and disruptions during the COVID-19 pandemic. As suggested by the reviewed literature, these pressures and disruptions may strain individuals’ relations with others, yet they may also lead to greater solidarity and support. Accordingly, I entertain two alternative hypotheses: that the quality of social relations has worsened during the pandemic (hypothesis 1a) and that it has improved (hypothesis 1b). Importantly, the direction and strength of the social repercussions of pandemic-induced challenges may vary across different categories of social actors and corresponding relations. Therefore, I look separately at the quality of relationships with different types of social actors, who represent a range of social significance. I start with women’s relations with their marital partners. Then, I consider women’s relations with their non-coresident children, typically late adolescents and young adults. Next, I look at women’s relations with their other relatives, broadly defined. I then move beyond the family and kinship circle. Because in rural sub-Saharan Africa and similar settings, religious involvement plays a central role in many women’s social lives outside the family circle (Pew Research Center 2016) and because this involvement may have been affected by lockdown policies, especially restrictions on religious service attendance, I look at women’s relations with members of their religious congregations. Finally, guided by the evidence on the importance of neighborhood cohesion and solidarity for mental health and general well-being (e.g., Ivory et al. 2011; Ruiz et al. 2019), I consider women’s relations with their neighbors. Although individual connections may vary within each of the multiple-actor categories, I expect, in general, to find greater changes in relationship quality with closest actors (i.e., family and kin).

I then link perceived changes in the quality of women’s social relations to the chosen general markers of subjective well-being: women’s overall life satisfaction and their expectations for improvement in their household’s conditions in the near future (which I also label as near-future optimism). I hypothesize that the pandemic-time changes in the quality of social relations are positively associated with life satisfaction (hypothesis 2) and with near-future optimism (hypothesis 3), regardless of women’s sociodemographic background and the economic impact of the pandemic on their households. However, because these associations may vary depending on the nature of social ties, I also anticipate these associations to be stronger for changes in the quality of relations with generally closer social actors, such as family members and kin, compared to coreligionists and neighbors.

## Context

The data for this study come from rural areas of the Gaza province of the Republic of Mozambique, an impoverished sub-Saharan nation with an annual gross national income per capita of U.S. $480 (World Bank 2022). The setting is typical of rural Africa in many respects. The local society is traditionally patrilineal and predominantly Christian, with a high level of religious involvement (Agadjanian and Yabiku 2015). Marriage is normatively virilocal and bridewealth based, yet bridewealth payments are often delayed or bypassed altogether as marriage becomes increasingly informalized (Chae, Agadjanian, and Hayford 2021). Despite the growing informalization of marriage and rising marital instability, marriage remains essential for rural women’s social identity and economic security (Tvedten, Paulo, and Tuominen 2010). Polygyny is common despite Christian churches’ nominal opposition to it (Agadjanian 2020). Fertility is high and is an important marker of women’s social status. Health and life expectancy of the local population has been greatly affected by the HIV/AIDS pandemic, with almost a quarter of adult Gaza residents estimated to be HIV positive (Ministry of Health 2019:5). The mainstay of the local economy is subsistence agriculture, with most farm work performed by women. Men’s labor migration, primarily to neighboring South Africa but also to Mozambique’s capital, Maputo, has long been a household risk diversification strategy (De Vletter 2007). Local employment opportunities outside subsistence farming are typically limited to petty trade and small crafts (Agadjanian, Hayford, and Oh 2021).

Like other parts of the Global South, Mozambique has been greatly affected by the COVID-19 pandemic. Although the true scale of infection and corresponding mortality in the country has been questioned, as in the rest of the sub-Sahara, because of the lack of reliable data, most of the pandemic’s socioeconomic impact, again, as elsewhere in the subcontinent, was driven by the lockdown and other restrictive policies introduced by the government in early April 2020 and resulting supply chain and employment disruptions. The COVID-19 restrictions also negatively affected migration and mobility, health service use, schooling, church attendance, and other collectivized activities. Most of those restrictions were removed by the middle of 2021.

## Data and Method

### Data

I use data from the Bridge COVID-19 (BC19) phone survey conducted in late 2021 and early 2022. The BC19 survey was part of the longitudinal Men’s Migration and Women’s Lives (MMWL) panel study. The first wave of the MMWL panel was conducted in 2006 with a sample of 1,678 women aged 18 to 40 years, married to migrants or nonmigrants, residing in 56 villages in four districts of Gaza province. The subsequent waves were carried out in 2009 (wave 2), 2011 (wave 3), 2014 (wave 4), and 2017–2018 (wave 5), with all but a small fraction of wave 5 participants interviewed in person. The 2021–2022 BC19 survey focused mainly on the effects of the COVID-19 pandemic but also updated some key sociodemographic characteristics of participants. Although most wave 5 participants provided mobile phone numbers, given the very high frequency of number change (e.g., because of breakdown, loss, and theft of handsets or following mobile phone companies’ promotional sales), 572 women could be reached and interviewed in the BC19 survey. The interviews were carried out mainly in Changana, the area’s main language. The refusal rate in BC19, as in main MMWL waves, was less than 1 percent.

To assess any potential selection bias in the BC19 sample, I compared it with the participants who were interviewed in person in wave 5 and had access to mobile phones (their own or of other household members) on their educational level (number of school years completed) and on household material status (a six-level household assets scale) measured at wave 5. The average values for these two characteristics are very similar: mean number of years of schooling 3.20 (*SD* = 0.10) versus 3.15 (*SD* = 0.06) and mean asset score 2.43 (*SD* = 0.07) versus 2.39 (*SD* = 0.04) in the wave 5 and BC19 samples, respectively. I acknowledge, of course, that the BC19 sample, like the wave 5 subsample with access to a mobile phone, has higher average socioeconomic status than the wave 5 participants who did not have phone access.

The BC19 survey asked participants to evaluate changes in their households’ economic situation during the pandemic, using the following question: “Since the beginning of the COVID pandemic, has the economic situation of your household improved, worsened, or almost not changed?” The BC19 instrument also included a series of questions on possible changes in the quality of participants’ relations with their marital partners (if they were in marital partnerships, formalized or not), with their non-coresident children (if they had any such children), with other relatives, with coreligionists (if they were religiously affiliated), and with neighbors. For each of these categories, the following question was asked: “Since the beginning of the Covid pandemic, have your relations with [type of social actor(s)] improved, worsened, or almost not changed?” It should be noted that participants were not asked to link causally any possible changes in either their economic situation or in their social relations to the effects of the pandemic. Moreover, no time frame was directly specified, and it was left up to participants to define the starting point of the pandemic (I assume that most of them probably linked it with the imposition of the first national lockdown in April 2020). I also acknowledge that, except for the questions on relations with marital partner, the number of individuals in each relational categories may be potentially consequential for individual well-being during crises such as the COVID-19 pandemic (cf. Kovacs et al. 2021). Also, relations with different individuals in each of those categories may have changed in different directions (or may not have changed at all). Thus, the survey answers capture participants’ overall perceptions of possible changes in the quality of their relations with all individuals in each category, even though they may be disproportionately influenced by changes in relations with some and even one of them. I also acknowledge that membership in several of the chosen relational categories may overlap (e.g., some neighbors may also belong to a participant’s church), but I assume that the subjective allocation of social actors into such potentially overlapping categories reflects what the participant perceived as their primary relational identity.

The BC19 participants were asked about their overall life satisfaction, using the following standard question: “Thinking of your life in general, are you very satisfied, quite satisfied, a little satisfied, or not satisfied with your life now?” The survey also included the following question on participants’ expectations for possible changes in their households’ general living conditions: “Thinking of the future, in your opinion, within the next year, will the living conditions of your household improve, worsen, or stay about the same?” Unlike the previously presented question on changes in household “economic situation” (*matshamela ya ta xuma* in Changana) during the pandemic, household “living conditions” (*mahanyelo ya njangu* in Changana) is a broader and more inclusive term connoting household general well-being. Importantly, the questions were positioned in different parts of the questionnaire to minimize cognitive priming, anchoring, or carryover. The design and content of the BC19 survey was approved by the Institutional Review Board of the University of California - Los Angeles.

### Method

My first interest is in participants’ assessments of changes (or lack thereof) in the quality of social relations since the onset of the pandemic. I model separately the reported changes for relations with marital partner, non-coresident children, other kin, coreligionists, and neighbors. Each of these outcomes is coded as a three-level ordinal scale: worsened (coded −1), have not changed much or at all (0), or improved (+1). I relate these dynamics in the social relationship quality to perceived changes in the household economic situation after the pandemic started. Given that such changes, if reported, were predictably toward worsening, I created a binary indicator of worsening of household economic situation: the economic situation has worsened (coded 1) versus has remained more or less the same or improved (coded 0).

I fit a series of multivariable ordinal regression models predicting changes in the quality of social relations in each of the five categories, with the worsening of household economic situation being the main covariate. These models control for participant’s age (ranging between 34 and 56 years), the total number of her living biological children, her education (years of completed schooling), and her current engagement in income-generating work outside subsistence agriculture (1 = works, 0 = does not work). All the models distinguish monogamously married women from polygynously married ones. All the models, except the one for relations with marital partner, include nonmarried (divorced or widowed) women as a separate category. Because the economic impact of the pandemic and its social implications may vary by household prepandemic conditions, all models also control for household material background derived from the wave 5 data. This variable is a six-level scale based on household possession of such items as a framed bed, radio, TV set, bicycle, motorcycle, water tank, and plough.

Next, I look at participants’ overall life satisfaction. Following the response options in the corresponding BC19 question, this outcome is operationalized as a four-level scale (1 = not satisfied, 2 = a little satisfied, 3 = quite satisfied, and 4 = very satisfied), and ordered logit models are used to predict it. The main predictor is change in relationship quality since the beginning of the pandemic, and separate models are fitted for each relations category. To estimate a net association of changes in the quality of relations with life satisfaction, I include the same covariates as described above and add participant’s self-rated physical health (1 = bad/so-so, 2 = good, 3 =excellent), as health is an important correlate of subjective well-being (Steptoe 2019). The models also control for life satisfaction as reported in wave 5. All the models control for the worsening of the household economic situation during the pandemic.

Finally, I consider participants’ expectations for their households’ general conditions in the next year (i.e., near-future optimism). Because such expectations inevitably involve considerable uncertainty, I include “not sure” responses in the analysis. Accordingly, I group all the responses into three categories: expects household conditions to definitely worsen in the next year (−1), expects them remain more or less the same or unsure whether or how they will change (0), and expects them to definitely improve (+1). I fit a series of ordinal logistic regression models, with changes in the quality of category-specific social relations being the main predictor. The models include the same controls as the life satisfaction models (except for life satisfaction at wave 5). Again, I reiterate that the proposed analyses do not assert causal connections between the predictors and outcomes, and the statistical effects reported in the following section should be interpreted accordingly. The distribution of all the variables used in the analysis is shown in Table 1.

**Table 1.** Descriptive Statistics, Bridge COVID-19 Survey, Mozambique.

Variable	Value
Change in quality of relations with (scale: −1 to +1; mean, *SD*)	
Marital partner (*n* = 457)	−.03 (.02)
Non-coresident children (*n* = 375)	−.07 (.02)
Other kin (*n* = 572)	−.10 (.02)
Coreligionists (*n* = 543)	−.09 (.02)
Neighbors (*n* = 572)	−.05 (.01)
Life satisfaction (scale: 1 to 4; mean, *SD*)	2.72 (.04)
Expected change in household general conditions in next year (scale: −1 to +1; mean, *SD*)	.10 (.03)
Household economic situation has worsened since the COVID-19 pandemic began (%)	53.32
Age (years; mean, *SD*)	43.20 (.26)
In monogamous marriage (%)	61.89
In polygynous marriage (%)	18.01
Divorced or widowed (%)	20.10
Number of biological children (mean, *SD*)	4.84 (.08)
Years of school completed (mean, *SD*)	3.15 (.06)
Works outside subsistence farming (%)	28.15
Self-rated health (scale: 1 to 3; mean, *SD*)	2.06 (.03)
Wave 5 material assets score (scale: 0 to 5; mean, *SD*)	2.39 (.04)
Life satisfaction at wave 5 (scale: 1 to 4; mean, *SD*)	2.71 (.03)
*n* (unless otherwise noted)	572

## Results

As can be seen in Table 1, slightly more than half of the BC19 survey participants, 53.3 percent, said that the economic situations of their households had worsened since the beginning of the pandemic. Table 1 also shows the means of participants’ standing on the three-point scale (worse, same, or better) of changes in relations with five categories of social actors. In all five categories, we can see a tendency toward deterioration since the start of the pandemic, the largest changes being in relationships with other relatives and the smallest in the relationship with the marital partner. However, if we break the sample down by whether the household economic situation worsened or not since the onset of the pandemic, we observe considerable variation across and within the subcategories. This variation is displayed in Table 2. Table 2 shows a particular stark contrast in changes in the quality of relations with marital partners and with non-coresident children: women who reported worsening of their household economic situations are also more likely to report considerable deterioration of their relations with marital partners and children, while the average changes in these relations tend in the positive direction for women whose households’ economic situations have not worsened. The contrast for changes in the quality of relations with other relatives is also quite salient, although unlike the previous two categories, these relations have deteriorated in both compared subgroups. Deterioration also characterizes relations with coreligionists and neighbors among participants who experienced economic worsening and those who did not, although for both categories of relations, the change is more pronounced among the former.

**Table 2.** Bivariate Association between Worsening of Household Economic Situation and Changes in Social Relationship Quality since the Beginning of the COVID-19 Pandemic, Bridge COVID-19 Survey, Mozambique.

Change in Quality of Relations with	Economic Situation Has Worsened since the Pandemic Began	Economic Situation Has Not Worsened since the Pandemic Began
Marital partner	−.10 (.04)	.04 (.03)
Non-coresident children	−.15 (.04)	.02 (.03)
Other kin	−.15 (.03)	−.05 (.03)
Coreligionists	−.11 (.03)	−.06 (.03)
Neighbors	−.08 (.02)	−.03 (.02)
*n*	305	267

*Note*: Data are expressed as mean (*SD*); scale: −1 to +1.

To test for these associations net of other factors, I fit five multivariable ordinal regression models. To remind, the models control for key sociodemographic characteristics measured in the BC19 survey, as well as for household material assets measured at wave 5. The results, presented in Table 3 as odds ratios, conform to the bivariate patterns displayed in Table 2, indicating a significant negative effect of the household economic worsening on the change in quality of relations with marital partner and non-coresident children and also with other kin. In comparison, this association is not significant for changes in relations with coreligionists and with neighbors. These patterns generally align with hypothesis 1a, as well as with my expectation that the quality of relationship with closest social actors (i.e., family and kin) would be particularly sensitive to the economic consequences of the pandemic.

**Table 3.** Changes in Relationship Quality since the Onset of the Pandemic, Ordinal Logistic Regression, Odds Ratios, Bridge COVID-19 Survey, Mozambique.

	Quality of Relations with				
	(A) Marital Partner	(B) Non-coresident Children	(C) Other Kin	(D) Co-religionists	(E) Neighbors
Household economic situation has worsened since the pandemic began	.49 (−3.26)**	.40 (−3.49)**	.56 (−2.80)**	.81 (−1.08)	.68 (−1.52)
Age	1.03 (.13)	1.04 (1.78)^+^	1.02 (1.33)	1.01 (.77)	1.01 (.50)
Marital status (reference: in monogamous marriage)					
In polygynous marriage	.50 (−2.70)**	.76 (−.87)	.84 (−.65)	.70 (−1.38)	1.45 (1.10)
Not married (divorced or widowed)	NA	.90 (−.33)	1.14 (.48)	.99 (−.04)	1.20 (.53)
Number of biological children	.89 (−1.97)*	.94 (−.88)	.99 (−.19)	.99 (−.21)	.96 (−.60)
Years of school completed	1.12 (2.11)*	1.03 (.54)	1.11 (2.14)*	1.02 (.33)	.96 (−.59)
Works outside subsistence farming	1.43 (1.48)	1.16 (.53)	1.32 (1.23)	1.58 (2.08)*	1.15 (.49)
Wave 5 material assets score	.99 (−.08)	.92 (−1.07)	.91 (−1.51)	.91 (−1.66)	1.00 (.01)
Likelihood ratio χ^2^	29.56**	18.44*	16.95*	10.77	5.26
*n*	457	375	572	543	572

*Note*: Values in parentheses are *z* statistics. NA = not applicable.

+ *p* < .10. *p < .05. **p < .01.

For overall life satisfaction, 12.1 percent of participants were not satisfied with their lives, 28.9 percent were a little satisfied, 34.3 percent were quite satisfied, and 24.8 percent were very satisfied, with an average score of 2.7 on the 4-point (range = 1–4) scale (see Table 1). The corresponding ordinal logistic regression models predict the level of life satisfaction from changes in the quality of social relations. The results of category-specific models, displayed in Table 4, show a marginally positive net effect for change in the quality of relations with marital partner (odds ratio = 1.38, *p* < .10). Notably, it is the addition of self-rated health that considerably reduces the magnitude and significance of this effect. In comparison, changes in the quality of relations with non-coresident children and with other relatives each have highly significant positive net effects. In contrast, changes in the quality of relations with coreligionists and neighbors show no net association with life satisfaction. My hypothesis 2 is therefore conditionally supported for changes in relations with the closest social actors.

**Table 4.** Overall Life Satisfaction, Ordinal Logistic Regression, Odds Ratios, Bridge COVID-19 Survey, Mozambique.

	A	B	C	D	E
Change in quality of relations with					
Marital partner	1.38 (1.71)^+^				
Non-coresident children		1.93 (3.02)**			
Other kin			1.66 (2.90)**		
Coreligionists				1.14 (.86)	
Neighbors					1.13 (.52)
Household economic situation has worsened since the pandemic began	.61 (−2.78)**	.59 (−2.56)**	.62 (−2.99)**	.64 (−2.67)**	.59 (−3.25)**
Age	1.03 (1.71)^+^	1.04 (2.38)*	1.03 (2.76)**	1.04 (2.50)**	1.04 (2.87)**
Marital status (reference: in monogamous marriage)					
In polygynous marriage	.62 (−2.22)^*^	.64 (−1.78)^+^	.62 (−2.26)*	.63 (−2.08)*	.60 (−2.38)*
Not married (divorced or widowed)	NA	.35 (−3.93)**	.36 (−4.73)**	.39 (−4.16)**	.36 (−4.69)**
Number of biological children	.97 (−.63)	.95 (−.87)	.96 (−.93)	.98 (−.57)	.96 (−.93)
Years of school completed	1.01 (.31)	1.04 (.84)	1.06 (1.41)	1.05 (1.23)	1.07 (1.68)^+^
Works outside subsistence farming	1.32 (1.38)	.94 (−.27)	1.29 (1.42)	1.17 (.84)	1.30 (1.48)
Wave 5 material assets score	1.07 (1.15)	.99 (−.16)	1.05 (.91)	1.06 (1.04)	1.04 (.73
Self-rated health	3.88 (9.39)**	4.08 (8.66)**	4.10 (10.87)**	4.48 (10.99)**	4.04 (10.72)**
Life satisfaction at wave 5	1.01 (.15)	.96 (−.28)	.98 (−.22)	.93 (−.63)	.97 (−.24)
Likelihood ratio χ^2^	130.91**	136.26**	196.62**	184.38**	188.44**
*n*	453	371	565	536	565

*Note*: Values in parentheses are *z* statistics. NA = not applicable.

+ *p* < .10. *p < .05. **p < .01.

Finally, I look at women’s expectations for changes in their households’ general conditions in the next year. Overall, 26.2 percent of participants expected definite improvements in household conditions, 16.1 percent expected that these conditions would definitely get worse, and 57.7 percent did not have clear expectations of either negative or positive change, with an average score of 0.10 on the three-point (range = −1 to +1) scale (see Table 1). The results of the multivariable ordinal logistic regression models that test for an association between change in relationship quality in each relational category and short-term future expectations are shown in Table 5. In contrast to the life satisfaction tests, only the change in the quality of relationship with marital partner has a significant positive effect on near-future optimism (odds ratio = 1.59, *p* < .05). For none of the other four relational categories does this association reach statistical significance. Hence, hypothesis 3 receives very limited support. In addition, in ancillary tests, I ran the same models excluding the “unsure” cases. These models, although reducing the analytic samples, produce almost the same effects of the main predictors of interest as the presented models, illustrating the semantic proximity between the “will stay more or less the same” and “unsure” categories in participants’ highly subjective reflections on their future.

**Table 5.** Expectations of Changes in Household General Conditions in the Next Year (Near-Future Optimism), Ordinal Logistic Regression, Odds Ratios, Bridge COVID-19 Survey, Mozambique.

	A	B	C	D	E
Change in quality of relations with					
Marital partner	1.59 (2.39)*				
Non-coresident children		.95 (−.24)			
Other kin			.84 (−.93)		
Coreligionists				1.01 (.08)	
Neighbors					1.26 (.97)
Household economic situation has worsened since the pandemic began	.82 (−1.04)	.69 (−1.72)^+^	.68 (−2.21)*	.72 (−1.86)^+^	.71 (−2.04)*
Age	.99 (−.48)	.99 (−.82)	.99 (−.56)	.99 (−.50)	.99 (−.62)
Marital status (reference: in monogamous marriage)					
In polygynous marriage	1.89 (2.79)**	1.90 (2.41)*	1.73 (2.47)*	1.88 (2.72)**	1.73 (2.47)*
Not married (divorced or widowed)	NA	.42 (−3.02)**	.52 (−2.85)**	.55 (−2.50)*	.517 (−2.87)**
Number of biological children	1.07 (1.32)	1.07 (1.19)	1.00 (−.10)	1.00 (.02)	1.00 (−.02)
Years of school completed	1.03 (.70)	1.07 (1.34)	1.06 (1.29)	1.07 (1.59)	1.05 (1.22)
Works outside subsistence farming	1.48 (1.84)^+^	1.37 (1.36)	1.52 (2.24)*	1.46 (1.94)^+^	1.49 (2.15)*
Wave 5 material assets score	1.29 (4.28)**	1.21 (2.84)**	1.23 (3.82)**	1.23 (3.77)**	1.23 (3.91)**
Self-rated health	1.63 (3.56)**	1.47 (2.62)**	1.62 (3.97)**	1.67 (4.09)**	1.64 (4.06)**
Likelihood ratio χ^2^	53.40**	54.73**	77.33**	75.16**	77.41**
*n*	457	375	572	543	572

*Note*: Values in parentheses are *z* statistics. NA = not applicable.

+ *p* < .10. *p < .05. **p < .01.

Among the statistical effects of other covariates in the models predicting the psychosocial well-being outcomes, it is noteworthy that the worsening of household economic situation during the pandemic has a consistently significant negative association with life satisfaction. In comparison, this association is generally less pronounced in the near-future optimism models. At the same time, the prepandemic household assets level shows no association with life satisfaction, but it is a strong predictor of near-future optimism. For both life satisfaction and near-future optimism, self-rated health is a powerful statistical determinant. Not married participants are distinctly less satisfied with their lives and less optimistic about the future than married participants. Among the latter, curiously, polygynously married women tend to be less satisfied with their lives but more optimistic about the future than their monogamously married counterparts, net of other factors.

In supplementary explorations, I created a combined scale of overall change in the quality of social relations with different types of social actors, also adjusting for the (non)existence of such actors as marital partner, non-coresident children, or coreligionists. This adjusted overall change in social relationship quality scale therefore allows the inclusion of the entire sample in the analysis. I fitted a series of ordinal logistic regression models predicting participants’ positioning on this integrated scale using the same predictors as in the category-specific models. The results of these ancillary tests generally parallel those presented above. Thus, worsening of the household economic situation showed a strong net negative effect on the overall social relations quality. Net of the household economic worsening, changes in the overall quality of social relations were positively associated with life satisfaction but showed no statistically significant effect on near-future optimism. The full results of these ancillary tests are presented in the Appendix.

## Discussion and Conclusion

In this study, I sought to contribute to a better understanding of longer term relational and psychosocial consequences of the COVID-19 pandemic–triggered disruptions and pressures for one of the most vulnerable, yet also least studied, segments of the sub-Saharan population: middle-aged rural women. I focused on changes in the quality of their relations with different types of social actors and possible associations of these changes with women’s life satisfaction and near-future optimism. Although these two measures may not accurately reflect the objective reality (e.g., actual changes in employment, earnings, or frequency and intensity of social interactions), they are nonetheless real, critical, and consequential elements of women’s well-being in midlife (cf. Kim et al. 2021).

As my findings suggest, women’s individual assessments of the pandemic-time changes in the quality of their closest (at least in the normative sense) relations—those with marital partners, non-coresident children, and other relatives—have a strong negative association with the worsening of their household economic situation during the pandemic, net of women’s sociodemographic characteristics and of the household long-term economic foundation. In comparison, no association between economic worsening and changes in the quality of relations with coreligionists and with neighbors, generally more distant and probably more diverse sets of social actors, was detected. These findings illustrate how the relations with generally more intimate social actors, which are typically more engaging and intense than those with more distanced ones, may be more subjectively sensitive to external shocks and fluctuations, such as those caused by pandemic-related disruptions and tensions (cf. Jace and Makridis 2021; Prime et al. 2020; Shepherd 2022).

When I examined the association of changes in relationship quality with overall life satisfaction, I also found this association to be strongest for relations with individuals in the generally more connected social categories: marital partner, non-coresident children, and kin. Interestingly, change in the quality of relations with marital partners showed a relatively weak and marginally significant net effect, as much of that effect was mediated by woman’s self-rated health. Of course, as with other analyses in this study, I cannot assert a causal direction of the association between health and marital relationship quality, as the latter may be affected by the former.

For what I labeled as near-future optimism, I did not find any significant connection with perceived changes in the quality of most social ties, possibly implying that such changes may not have a strong prospective connection with women’s subjective well-being. One notable exception, however, was change in the quality of relationship with marital partner, which showed a statistically significant positive association with expectations for short-term changes in household conditions even after controlling for other factors. Of the five categories of social ties, relations with marital partners are, I argue, most consequential for women’s perceptions of their economic and social security and stability, especially in such a deeply patriarchal setting, where women’s well-being is highly contingent on their marital status and relationship (cf. Tvedten et al. 2010). It is not surprising, therefore, that in all the other models, which included not married (divorced and widowed) women, these women were less optimistic about the prospects of their households and less satisfied with their lives, compared with married women, regardless of the economic impact of the pandemic or their background characteristics.

I acknowledge the limitations of my analysis. The BC19 data do not allow estimation of the sizes of personal networks, the degree of connectedness, and the intensity of ego’s interactions with individual network members, which are likely to change following crises such as the COVID-19 pandemic (see e.g., Forgette et al. 2009; Kovacs et al. 2021; Krendl and Perry 2020; Phan and Airoldi 2015). Also, my study cannot capture temporal variations in specific social relations and the resulting complexity of the social fallout of the pandemic, especially as its effects linger but also increasingly intersect with challenges of soaring inflation and deepening economic and nutritional insecurities in Mozambique and across the Global South. Yet my findings shed important light on how major epidemiological and societal shocks may affect the social fabric and, in turn, imprint individual psychosocial well-being, especially among such vulnerable groups as middle-aged women in impoverished patriarchal settings. Further research and corresponding policy interventions aimed at optimizing women’s social and emotional trajectories and improving their experiences across the life course should pay systematic and careful attention to these complex yet highly consequential dynamics.

## Supplemental Material

sj-docx-1-srd-10.1177_23780231231171868 – Supplemental material for The COVID-19 Pandemic, Social Ties, and Psychosocial Well-Being of Middle-Aged Women in Rural Africa Click here for additional data file. Supplemental material, sj-docx-1-srd-10.1177_23780231231171868 for The COVID-19 Pandemic, Social Ties, and Psychosocial Well-Being of Middle-Aged Women in Rural Africa by Victor Agadjanian in Socius
