# Supplementary material for: The COVID-19 Pandemic, Social Ties, and Psychosocial Well-Being of Middle-Aged Women in Rural Africa
Source: Socius. 2023 May 20;9:23780231231171868. doi: 10.1177/23780231231171868 (PMC10201067; doi:10.1177/23780231231171868)
Supplement: sj-docx-1-srd-10.1177_23780231231171868 – Supplemental material for The COVID-19 Pandemic, Social Ties, and Psychosocial Well-Being of Middle-Aged Women in Rural Africa [file sj-docx-1-srd-10.1177_23780231231171868.docx]

**APPENDIX**

Table A.1. Change in overall social relationship quality; 2. Life satisfaction; 3. Expectations of changes in household conditions in next year (near-future optimism), ordinal logistic regressions, odds ratios, BC19 Survey, Mozambique

|  | 1. Change in overall relations quality | 2. Life satisfaction | 3. Near-future optimism |
| --- | --- | --- | --- |
| Change in overall quality of social relations | n/a | 1.47  (3.19)^**^ | 1.03  (0.20) |
|  |  |  |  |
| Household economic situation has worsened since the pandemic began | 0.54  (-3.73)^**^ | 0.63  (-2.84)^**^ | 0.70  (-2.06)^*^ |
|  |  |  |  |
| Age | 1.02  (1.59) | 1.04  (2.66)^**^ | 0.99  (-0.61) |
| Marital status [Ref: in monogamous marriage] |  |  |  |
| In polygynous marriage | 0.59  (-2.45)^*^ | 0.64  (-2.07)^*^ | 1.75  (2.50)^*^ |
| Not married (divorced or widowed) | 0.95  (-0.26) | 0.36  (-4.67)^**^ | 0.52  (-2.86)^**^ |
| Number of biological children | 0.95  (-1.32) | 0.97  (-0.79) | 1.00  (-0.05) |
| Years of school completed | 1.05  (1.32) | 1.06  (1.49) | 1.05  (1.19) |
| Works outside subsistence farming | 1.53  (2.35)^*^ | 1.23  (1.16) | 1.49  (2.15)^*^ |
| Wave 5 material assets score | 0.93  (-1.41) | 1.05  (0.90) | 1.23  (3.89)^**^ |
| Self-rated health | n/a | 4.10  (10.86)^**^ | 1.62  (4.00)^**^ |
| Life satisfaction at Wave 5 | n/a | 0.97  (-0.27) | n/a |
|  |  |  |  |
| Likelihood ratio chi-square | 31.35^**^ | 198.46^**^ | 76.51^**^ |
| *N* | 572 | 572 | 572 |

Notes: *z* statistics in parentheses; significance level: ^+^ *p* < 0.10, ^*^ *p* < 0.05, ^**^ *p* < 0.01.
